# Control Potential of Multiple Nucleopolyhedrovirus (SfMNPV) Isolated from Fall Armyworm in Nigeria (West Africa)

**DOI:** 10.3390/insects15040225

**Published:** 2024-03-26

**Authors:** Ghislain T. Tepa-Yotto, Ouorou Kobi Douro-Kpindou, Précieux Sèna Bonaventure Koussihouédé, Abissi Marc Adjaoké, Jeannette K. Winsou, Ghislain Tognigban, Manuele Tamò

**Affiliations:** 1 Biorisk Management Facility (BIMAF), International Institute of Tropical Agriculture (IITA-Benin), Cotonou 08-01000, Benin; d.kpindou@cgiar.org (O.K.D.-K.); precieuxkouss@gmail.com (P.S.B.K.); j.winsou@gmail.com (J.K.W.); m.tamo@cgiar.org (M.T.); 2 Ecole de Gestion et de Production Végétale et Semencière (EGPVS), Université Nationale d’Agriculture (UNA), Kétou 43, Benin; 3 Ecole Doctorale des Sciences Agronomiques et de l’Eau (EDSAE), Université Nationale d’Agriculture (UNA), Kétou 43, Benin; marc.adjaoke@gmail.com (A.M.A.); tognigbanghislain@gmail.com (G.T.)

**Keywords:** entomopathogen, virulence, concentration, host plant, cannibalism

## Abstract

**Simple Summary:**

The fall armyworm (FAW) *Spodoptera frugiperda* is an invasive pest that causes damage to several crops including maize, its preferred host plant. Initial outbreaks of the pest on the African continent were recorded in early 2016. A variety of management options were explored in Africa to control the pest. The use of biopesticides and biological control was one of them. The current study aimed at measuring the susceptibility of FAW larvae to an entomopathogenic virus, SfMNPV, isolated from Nigeria (West Africa). The findings proved that the new SfMNPV isolate from Nigeria (SfMNPV-KA1) is more effective than its exotic counterpart from Argentina (SfMNPV-ARG), paving the way for bioinsecticide use to control the fall armyworm in Africa.

**Abstract:**

The fall armyworm (FAW) *Spodoptera frugiperda* (Lepidoptera, Noctuidae) has now become an invasive pest of global concern. The pest was first detected in Central and Western Africa in early 2016. Sustainable management options explored by stakeholders during early FAW invasion in Africa included the use of biopesticides and biological control. The current study aimed to compare the susceptibility of FAW larvae to SfMNPV with the assumption that the virus isolated from FAW populations in Africa has higher virulence compared with an isolate from Argentina (SfMNPV-ARG). We also hypothesized that host plant plays a role in SfMNPV efficacy and that cannibalism mediates horizontal and vertical transmission of the virus. This work provides pioneering data on the virulence of the new SfMNPV isolate from Nigeria (SfMNPV-KA1), which proved more effective than its exotic counterpart from Argentina (SfMNPV-ARG). The host plant effect made a significant difference between maize and onion with more FAW death in the larvae fed with contaminated onion 5 days post treatment. The study demonstrates and discusses the effect of cannibalism on virus transmission.

## 1. Introduction

The fall armyworm (FAW) *Spodoptera frugiperda* (Lepidoptera, Noctuidae) has now become an invasive pest of global concern. It is native to the Americas. The pest was first detected in Central and Western Africa in early 2016 [1]. Fall armyworm was further found and confirmed in the whole Southern African mainland (except Lesotho), Madagascar and Seychelles (Island State). Within two years post-detection, it had spread across almost all of sub-Saharan Africa. By November 2019, FAW was also reported and confirmed in Sudan and Egypt (Africa) and Yemen (West Asia). Additional reports were received in several Asian countries including India, Bangladesh, Sri Lanka, Thailand, Myanmar, China, Indonesia, the Philippines, Laos, Malaysia, Vietnam, Cambodia, the Republic of Korea, and Japan. Between February and May 2020, it was confirmed in Australia, Mauritania, Timor-Leste, and the United Arab Emirates. Later, in 2020, it was found in Jordan, Syria, and Papua New Guinea. In January 2021, New Caledonia confirmed FAW and by April it had invaded the Canary Islands of Spain in Europe. In 2023, it was also confirmed in Türkiye and Cyprus. The pest was reported in Vanuatu (Oceania) and Romania (Europe) in 2024. As of today, FAW has spread from the Americas to nearly 100 countries across the globe [2].

**Figure 1:**
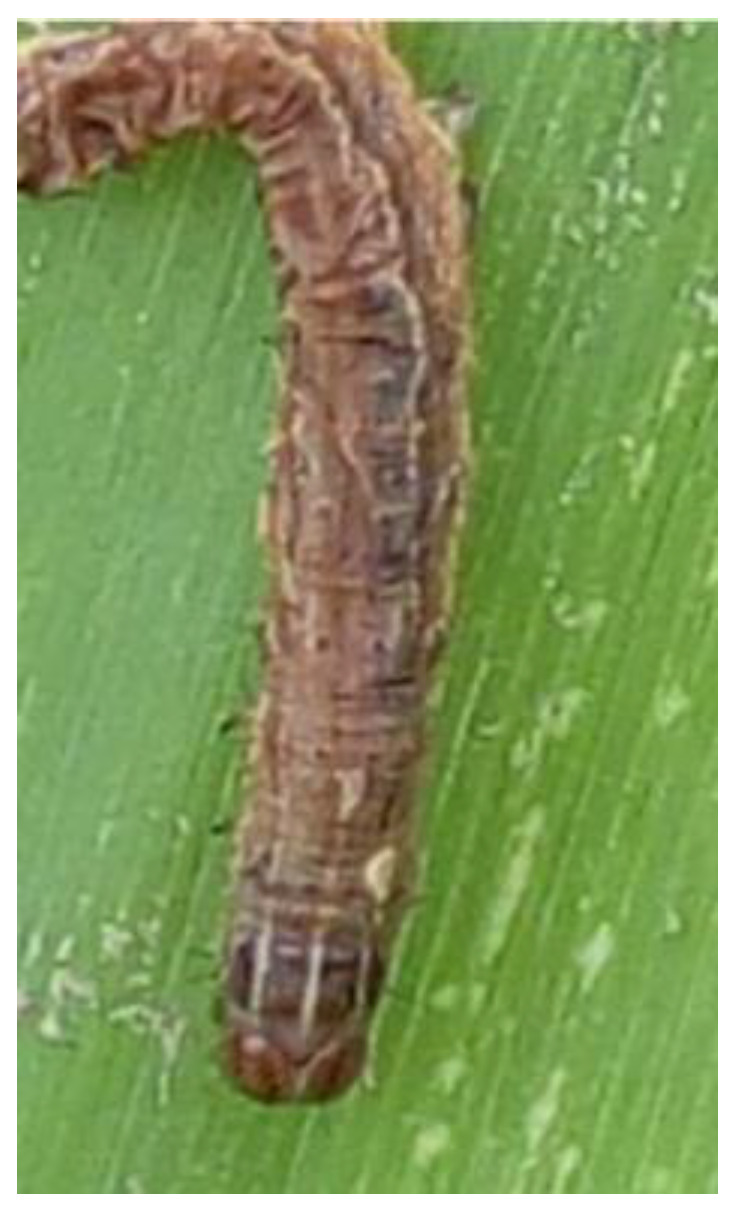
SfMNPV-KA1 infection on fall armyworm caterpillar isolated in Checheyi, Kwali-Abuja, Nigeria. Photo by Ghislain T. Tepa-Yotto.

Sustainable management options explored by stakeholders upon early FAW invasion in Africa [3] include biopesticides and biological control [4]. It is commonly accepted that biopesticides consist of microbial control agents, insecticidal plant extracts, or essential oils [5,6,7,8,9]. Viral entomopathogens are often catalogued as associated with the highest potential for development into bioinsecticide products due to specificity, high host virulence, and highest safety to the environment and vertebrates [10]. Most of them belong to the Baculovirus group. Two types of Baculovirus have been promoted for the control of *S. frugiperda*, namely, granulovirus (SfGV) (Betabaculovirus) and multiple nucleopolyhedrovirus (SfMNPV) (Alphabaculovirus). SfMNPV is specific to FAW larvae and thus has greater potential for use in pest management [11]. In nature, the pest is infected orally by ingesting contaminated food (usually maize leaf) [12]. Once ingested, the polyhedral inclusion bodies (PIB) dissolve in the alkaline midgut, releasing infective virions. These virions infect the midgut epithelium cells and multiply in the nucleus. Furthermore, the virus propagates to the host body cavity and infects other tissues such as adipose tissue, epidermal, tracheal matrix, salivary glands, Malpighian tube, and blood cells. This can cause host death after 6 to 8 days post ingestion. A caterpillar infected with nucleopolyhedrovirus declines in feeding ability and eats only 7% of the food normally consumed by a healthy caterpillar [4,13]. The symptoms of Baculovirus infection include the appearance of blemishes and yellowing of the skin. An infected larva typically moves to the upper parts of the plant and, upon death, hangs its head downward, with a few prolegs still attached to the host plant. The dead larvae are soft, darkish, and crumble easily to release polyhedrons, which further spread the virus (Figure 1) [4].

Parallel to expeditions in the Americas with the goal of selecting promising SfMNPV isolates to control FAW in Africa, a question that emerged on potential microbial activity in pest populations that entered the continent was whether the pest travelled while festering SfMNPV in its latent phase. This signaled field scouting to find FAW larvae killed by entomopathogenic viruses and fungi. To date, a few viral and fungal entomopathogens have been isolated from FAW eggs and larvae in the field [14,15,16,17]. However, how effective the entomopathogens are remains a researchable question. The current study aimed to compare the susceptibility of FAW larvae to SfMNPV on the assumption that the virus isolated from FAW populations in Africa is more virulent compared to an older exotic counterpart that was isolated in another ecology [18,19]. We also hypothesized that the host plant plays a role in SfMNPV efficacy [20,21]. Finally, because FAW is a cannibalistic species at its larval stage, particularly from third instars onward, we hypothesized that cannibalism mediates horizontal and vertical transmission of the virus [22,23,24].

## 2. Materials and Methods

### 2.1. SfMNPV Isolate Collection and Identification

*Spodoptera frugiperda* Multiple Nucleopolyhedrovirus (SfMNPV) was imported from Argentina (SfMNPV-ARG) and quarantined at the International Institute of Tropical Agriculture (IITA), Benin station, for testing purposes against FAW during initial pest outbreaks in 2016 [1].

A new SfMNPV isolate from Nigeria (SfMNPV-KA1) was obtained from two infected FAW second- and fourth-instar larvae (Figure 1) that were collected in the Sudan-Guinea savannah on 9 September 2017 from a maize field in Checheyi village (Kwali-Abuja, Nigeria [8°579360 N, 7°299130 E]) (collected by Ghislain T. Tepa-Yotto). The sampled maize field was planted on 8 August 2017 and remained unsprayed until the collection date. The sample was transferred to the International Institute of Tropical Agriculture (IITA), Benin station, for laboratory multiplication (import permit 130/DIP-09/2017/SPVCP and quarantine inspection report and multiplication authorization 0029/IP-11/2017/SPVCP/CQF-A) and subsequent identification and genome sequencing at Julius Kühn Institute (JKI) (Darmstadt, Germany) [16].

### 2.2. Experimental Sprouting Maize and Onion

This operation consisted first of germinating maize seeds (variety EVDT) in plastic plates. Water was supplied every morning (08:00 a.m.) and evening (04:00 p.m.) until seedlings emerged. Watering frequency was reduced to once a day for older sprouts. Onion (variety SAFARI) was sown in plastic pots and watered similarly to maize. Three weeks were enough to obtain experimental onion leaves.

### 2.3. Laboratory Inoculum Production

All experiments were conducted at the Biorisk Management Facility (BIMAF) hosted by the International Institute of Tropical Agriculture (IITA), Benin station. Experimental conditions were 26 ± 1 °C, 70% RH, and 12 h photophase. Sprouting maize of the variety EVDT was produced to feed FAW larvae using a routine insect mass-rearing protocol [25].

SfMNPV-ARG (Argentina) and SfMNPV-KA1 (Nigeria) isolates were mass-produced using an in-house inoculum production procedure. This consists of an in vivo production of the inoculum as per Cherry et al. [26]. A sterile artificial diet was first poured into plastic trays. The medium was then sprayed with a dose of SfMNPV equivalent to 1 × 10^6^ PIB of the microbe. The contaminated food was offered ad libitum to starved larvae and left for a week at 26 °C for the NPV to multiply in the larvae. Successfully infected larvae were selected when incubation ended, and virions were extracted by crushing the infected larvae. The crushed material was filtered and centrifuged to obtain a pure viral solution. The different solutions were prepared at various concentrations, namely, 10^5−8^ polyhedral inclusion bodies (PIB) per mL.

### 2.4. Experiment 1: Virulence of the Newly Isolated SfMNPV-KA1

The virulence of SfMNPV-ARG and SfMNPV-KA1 isolates was compared on FAW first and third instar larvae. The two SfMNPV isolates were tested at four concentrations (10^5−8^ PIB per mL) using sprouting maize leaves soaked in the viral solutions for 15 min. The control concentration consisted of larvae fed with uncontaminated leaves. All experimental larvae were starved for six hours to increase feeding ability before they were served with the host plant. Larval mortality was monitored daily to count dead larvae with symptoms of virus infection within fifteen (15) days post treatment with SfMNPV. In addition, pupal formation and adult emergence were quantified. In total, 500 larvae of each experimental instar of FAW (first and third) were used for the first bioassay.

### 2.5. Experiment 2: Host Plant Effect on the Newly Isolated SfMNPV-KA1 Virulence

Two host plants, namely maize and onion, were used in the experiment on first instar larvae of FAW. The effect of SfMNPV-KA1 isolate was evaluated using experimental host plant leaves contaminated with the virus. To this purpose, first instar larvae of FAW were selected from the rearing facility. Each treatment consisted of twenty (20) larvae with five (5) replicates. Experimental larvae were placed individually in a sterile 5 cm-diameter Petri dish. Host plant (either maize or onion) leaves were contaminated by soaking them in the viral solution at two different concentrations, 10^5^ and 10^8^ polyhedral inclusion bodies (PIB) per mL; control treatments comprised FAW larvae fed with uncontaminated maize and onion host plants. The larvae were starved for six hours before the experiment started. The food (maize/onion contaminated with SfMNPV-KA1) was provided to FAW larvae, summing the experimental larvae to a total of 600 larvae used including control treatments.

### 2.6. Experiment 3: Cannibalism-Mediated Horizontal and Vertical Effect of the Newly Isolated SfMNPV-KA1

In this study, all experimental larvae (cannibal and prey) were starved for 6 h before bioassays. Second instar preys of FAW were first fed for 24 h with SfMNPV-KA1-contaminated sprouting maize leaves before exposure to uninfected conspecific fourth instar cannibals. Prey were infected with two different concentrations of SfMNPV-KA1 (10^5^ and 10^8^ polyhedral inclusion bodies (PIB) per mL). Control treatments consisted of uninfected prey exposed to uninfected cannibals (Video S1 A–D). A total of 20 pairs (prey against cannibal) were arranged per treatment with five replicates. The above described procedure was applied to four experimental subsets to measure: (i) cannibal consumption speed of prey infected with different PIB concentrations of SfMNPV-KA1; (ii) cannibal body part attack target on prey infected with two different PIB concentrations of SfMNPV-KA1; (iii) cannibal larval mortality and pupal and adult emergence post consumption of prey infected with two different PIB concentrations of SfMNPV-KA1; (iv) female cannibal oviposition rate post consumption of prey infected with different PIB concentrations of SfMNPV-KA1. Unresponsive larval pairs were discarded from the experiments. For the first experimental subset (i) a stopwatch was used to record the response time of each cannibal–prey pair, meaning the duration of attack/defense. Cannibal–prey reaction within 0–5 min, 6–10 min, 11–15 min and >15 min was coded as fast, moderate, slow and very slow speed responses, respectively. The second experimental subset (ii) consisted of careful observations to determine prey body part attacked and consumed by cannibal: prey head, abdomen or tail. In the third experimental subset (iii) daily counting of cannibal mortality rate, pupal formation and adult emergence was performed for a period of 15 days. Finally, the fourth experimental subset (iv) consisted of monitoring mated cannibal females to count the number of egg masses and the total number of eggs laid. The survival rate of hatching larvae was also followed for 15 days. A total of 600 experimental larvae were used to initiate these bioassays.

### 2.7. Data Analysis

All data were log-transformed before analysis to meet the assumptions of normality and equal variance. The data were then analyzed using a linear model analysis of variance (ANOVA) type II sum of squares with fixed effect factors. Only significant interactions between main effects were included in the models. Tukey’s post-hoc tests at the 5% significance level were used to examine differences among the groups, followed by pairwise comparisons [27].

## 3. Results

### 3.1. Experiment 1: Virulence of the Newly Isolated SfMNPV-KA1

**Figure 2:**
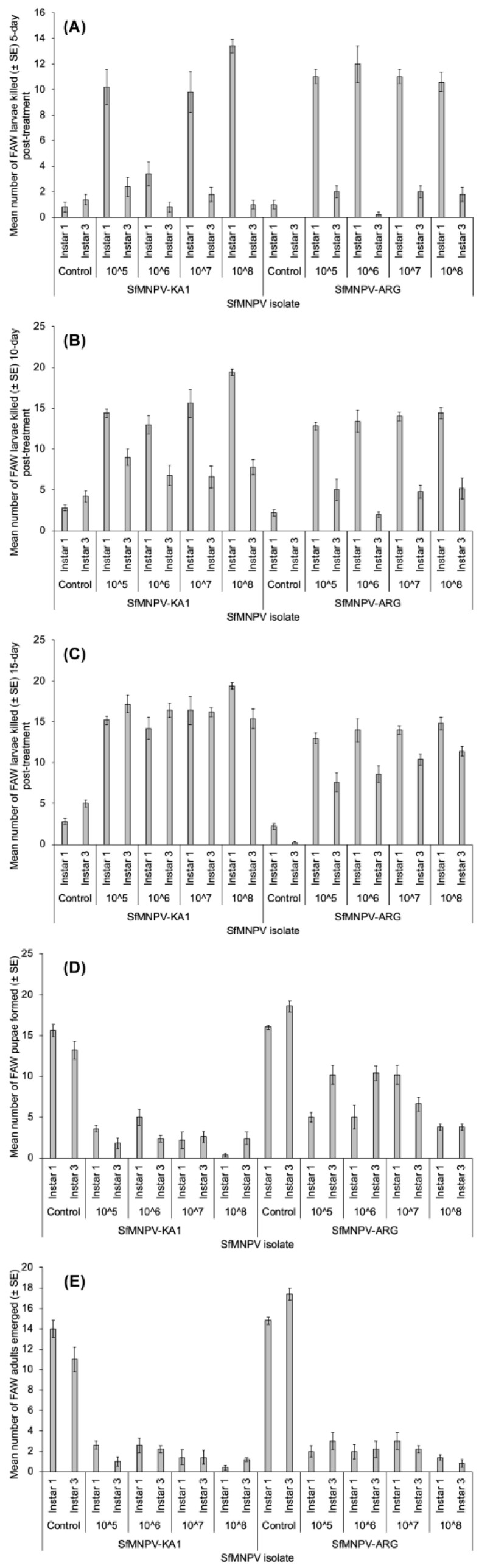
Effect of different concentrations (control and 10^5−8^ PIB × mL^−1^) of two isolates of SfMNPV (SfMNPV-KA1 and SfMNPV-ARG) on FAW first and third instars mortality 5 (**A**), 10 (**B**) and 15 days (**C**) post treatment, and on FAW pupae formed (**D**) and adults emerged (**E**) post treatment.

SfMNPV-KA1 killed more FAW larvae than SfMNPV-ARG, 10 days (*F*_1,88_= 36.406; *p* = 3.7 × 10^−8^) and 15 days (*F*_1,88_ = 36.406; *p* = 3.7 × 10^−8^) post treatment, although not at 5 days post treatment (*F*_1,80_ = 3.967; *p* = 0.049). Isolate concentration effect made a statistical difference in killing FAW compared to the control concentration (*F*_4,80_ = 44.586; *p* = 2.2 × 10^−16^). In almost all instances low concentrations were equally effective as higher concentrations. The first instars of FAW were more susceptible to the isolates compared to the older third instars (Figure 2; *F*_1,80_ = 443.719; *p* = 2.2 × 10^−16^). In all experiments, pupal and adult emergence was lower on larvae infected with SfMNPV-KA1 (*p* < 0.0001). At 5 days post treatment, all second-level interactions were significant, but only host instar and SfMNPV isolate effects interacted significantly at 15 days for larval mortality. Regarding pupal and adult emergence, almost all second-level interactions were significant except host instar × SfMNPV concentration for adult emergence.

### 3.2. Experiment 2: Host Plant Effect on the Newly Isolated SfMNPV-KA1 Virulence

**Figure 3:**
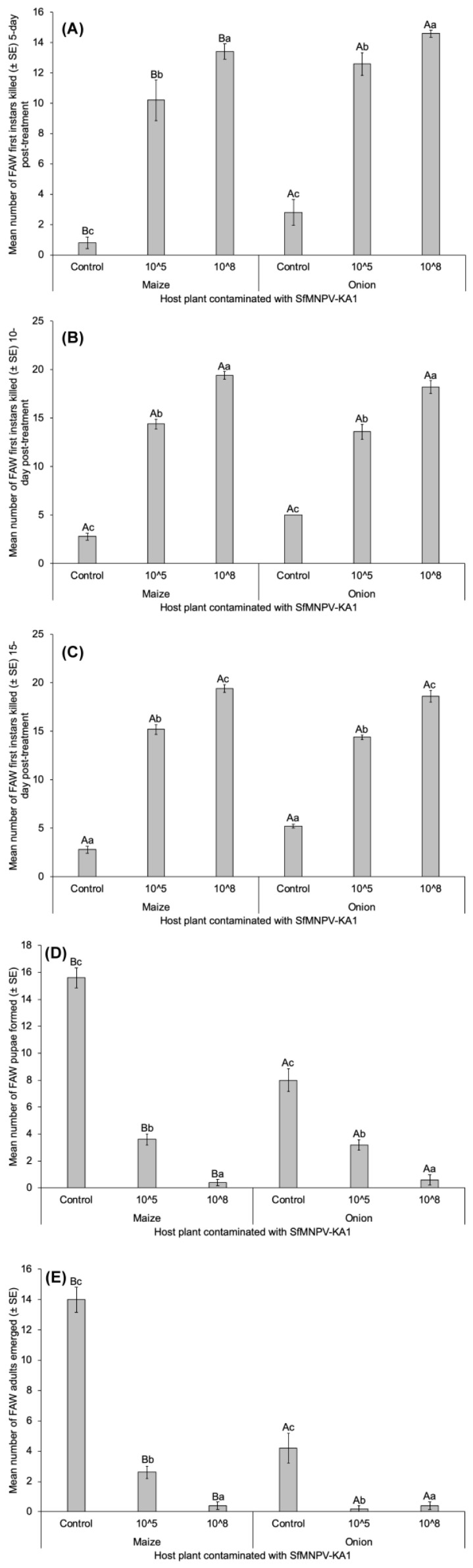
Effect of two host plants (maize and onion) on SfMNPV-KA1 isolate virulence to kill FAW first instar larvae 5 (**A**), 10 (**B**) and 15 day (**C**) post treatment; and on FAW pupae formed (**D**) and adults emerged (**E**) post-treatment. Means followed by same uppercase, and lowercase letters are not different between SfMNPV concentrations (control, 10^5^ and 10^8^ PIB × mL^−1^), and host plants (maize and onion), respectively (Tukey’s tests at the 5% level).

SfMNPV-KA1 killed more FAW larvae 5 days post treatment (*p* < 0.0001), but not beyond (10 and 15 days post treatment (*p* > 0.05)) (Figure 3). Host plant effect had a significant difference between maize and onion with more FAW deaths in larvae fed with contaminated onion 5 days post treatment (*p* = 0.005), but not at 10 and 15 days post treatment (*p* > 0.05). Almost all host plant (maize and onion) × SfMNPV concentration (10^5^ and 10^8^ polyhedral inclusion bodies (PIB) per mL) interactions were significant.

### 3.3. Experiment 3: Cannibalism-Mediated Horizontal and Vertical Effect of the Newly Isolated SfMNPV-KA1

#### 3.3.1. Cannibal Consumption Speed on Prey Infected with Two Different PIB Concentrations of SfMNPV-KA1

**Figure 4:**
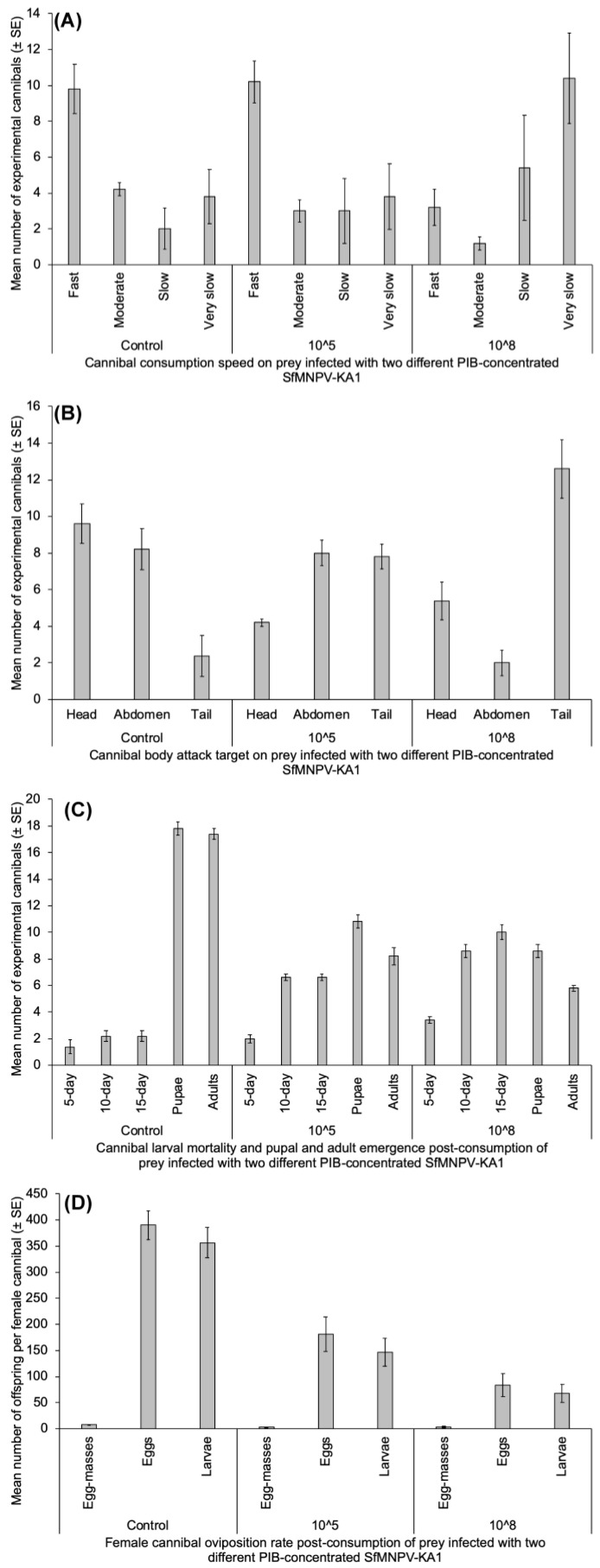
SfMNPV-KA1-infected prey effect on cannibalism and cannibal survival and fecundity; (**A**) Cannibal consumption speed of prey infected with two different PIB concentrations of SfMNPV-KA1; (**B**) Cannibal body part attack target on prey infected with two different PIB concentrations of SfMNPV-KA1; (**C**) Cannibal larval mortality and pupal and adult emergence post consumption of prey infected with two different PIB concentrations of SfMNPV-KA1; (**D**) Female cannibal oviposition rate post consumption of prey infected with two different PIB concentrations of SfMNPV-KA1.

Most cannibals had fast consumption speed of their prey independent of SfMNPV-KA1 concentration (*F*_3,48_ = 6.18; *p* = 0.001). The cannibal consumption speed of prey was not a function of prey infection status for different concentrations of SfMNPV-KA1 (*F*_2,48_ = 0.003; *p* = 0.996). However, the consumption speed of cannibals attacking prey infected with 10^8^ PIB × mL^−1^ decreased, relatively speaking, and the number of cannibals displaying this behavior was 3.2-fold higher than those with fast consumption speed (Figure 4A).

#### 3.3.2. Cannibal Body Part Attack Target on Prey Infected with Two Different PIB Concentrations of SfMNPV-KA1

When considering main effects, prey body parts (head, abdomen or tail) were equally attacked by cannibals (*F*_2,36_= 2.004; *p* = 0.149). However, the data demonstrated significant interaction between prey body part attacked and concentration of SfMNPV-KA1 (*F*_4,36_ = 23.88; *p* = 1.04 × 10^−9^), which is evidence of a cannibal decision to switch to a given prey body target based on the prey infection status. Most cannibals targeted the heads of non-SfMNPV-KA1-infected prey. By contrast, when infected with a high SfMNPV-KA1 concentration of 10^8^ PIB × mL^−1^, prey were more frequently attacked at their tails (Figure 4B).

#### 3.3.3. Cannibal Larval Mortality and Pupal and Adult Emergence Post Consumption of Prey Infected with Two Different PIB Concentrations of SfMNPV-KA1

Cannibal larval mortality post consumption of prey infected with two different PIB concentrations of SfMNPV-KA1 generally revealed statistical differences (*p* < 0.001). The mortality rates of cannibals that consumed prey infected with 10^8^ PIB × mL^−1^ were 2.4- to 4.5- and 1.3- to 1.7-fold higher than of the control and the cannibals killed after consumption of prey infected with 10^5^ PIB × mL^−1^, respectively (Figure 4C). In general, cannibal pupal formation and adult emergence post consumption of prey infected with the two different PIB concentrations of SfMNPV-KA1 also unveiled statistical differences (*p* < 0.0001) with highest rates among control cannibals (Figure 4C).

#### 3.3.4. Female Cannibal Oviposition Rate Post Consumption of Prey Infected with Two Different PIB Concentrations of SfMNPV-KA1

Female cannibal oviposition rate post consumption of prey infected with two different PIB concentrations of SfMNPV-KA1 (number of egg masses and total number of eggs laid) proved statistical differences (*p* < 0.001). The number of eggs laid by control female cannibals were 2.2- to 4.6- and 2.1- to 2.3-fold higher than by female cannibals that consumed prey infected with 10^8^ and 10^5^ PIB × mL^−1^, respectively (Figure 4D). Both female cannibal types previously exposed to prey infected with SfMNPV-KA1 (10^5^ and 10^8^ PIB × mL^−1^) gave birth to a statistically equal number of egg masses, but not an equal total number of eggs laid. Female cannibal progeny survival rate post consumption of prey infected with the two different PIB concentrations of SfMNPV-KA1 (number of larvae emerged/survived) displayed statistical differences (*F*_2,72_ = 35.78; *p* = 1.6 × 10^−11^). The number of larvae hatching from eggs of control female cannibals was 5.3- and 2.4-fold higher than of female cannibals that consumed prey infected with 10^8^ and 10^5^ PIB*mL^−1^, respectively (Figure 4D). Statistically, equal numbers of larvae developed from eggs laid by both female cannibal types previously exposed to prey infected with SfMNPV-KA1 at the two concentrations.

## 4. Discussion

To the best of our knowledge and the consulted literature, the new SfMNPV isolate from Nigeria (SfMNPV-KA1) was the first discovery of SfMNPV on the African continent [16]. SfMNPV-KA1 killed more FAW larvae than its exotic counterpart from Argentina (SfMNPV-ARG). Consistently, pupal and adult emergence rate was lower in SfMNPV-KA1. In addition, low isolate concentrations were frequently equally effective as higher concentrations, suggesting promising control potential of FAW in Africa using SfMNPV-KA1. In initial screenings, SfMNPV-ARG was found to be the most virulent exotic isolate compared to three others from the United States (SfMNPV-FX), Nicaragua (SfMNPV-NC) and Mexico (SfMNPV-UNAL) origins. The current study reports a significant SfMNPV-KA1 overperformance on SfMNPV-ARG, thereby exhibiting opportunities for bioinsecticide development. However, successful field application of baculoviral bioinsecticides is multifactor-dependent. These include and are not limited to virus virulence and prevailing climatic conditions, basically temperature, precipitation, humidity, and solar radiation. Nonetheless, other factors like plant growth stage, spray device, formulation used, and time of spray are also key to virus efficacy [4,28]. For instance, better efficiency of Baculovirus in FAW control is obtained when applied to maize plants at the 8- to 10-leaf early-whorl stage (2.5 × 10^11^ PIB per ha) on newly hatching larvae, applied once a week or in single application schemes [4]. Larval mortality of up to 97.2% can occur seven days after virus application. The spraying can be performed with the same equipment used for the application of a conventional chemical. However, it is recommended to use a fan nozzle (8004 or 6504) specifically for FAW [4]. Moreover, the more uniform the crop planting, the more successful the application. The insecticidal properties of SfMNPV can also vary between the maize strain and rice strain of the fall armyworm [29]. All the above-mentioned limiting factors on the success of baculoviral bioinsecticide led to investigations of enhanced effectiveness through its combination with biorationals or synthetic low-toxicity insecticides on a range of noctuid pests [30,31,32,33]. A dual-action bioinsecticide was also explored using mixed entomopathogens [34,35,36,37], but this was not always conclusive [38,39].

Significant second-level interactions could be explained by viral virulence’s dependence on PIB concentration and host instar. Indeed, first instars of FAW were more susceptible to the isolates compared to older third instars. This is not unusual with SfMNPV; younger larvae are most susceptible to the virus, while older ones develop resistance [4]. However, there are some data showing that older host instars may be suitable for studying synergism between two mixed baculoviruses [34]. From another perspective, the extent to which the discovered SfMNPV-KA1 can kill close genera/family counterparts is a researchable question.

There are just a few studies reporting on host plants affecting virus virulence [40,41]. FAW larvae were mostly killed when fed with contaminated onion compared to maize 5 days post treatment. This was surprising since the main host plant of FAW is maize. Conversely, it could indicate differences in PIB diffusion and spread through specific leaf tissues. This was partly confirmed by significant interactions between the host plant (maize and onion) and SfMNPV-KA1 concentration (10^5^ and 10^8^ polyhedral inclusion bodies (PIB) per mL). The findings inform host plant decision-making, particularly in the view of mass-producing SfMNPV using natural media. They also corroborate insect melanization-related enzyme activity associated with diets with low viral loads, as demonstrated in earlier works [42,43]. In addition, both Cisneros et al. and Gómez et al. [28,44] reported that improved formulations of SfMNPV with maize flour and 1% boric acid and microencapsulation are effective for the control of FAW. Furthermore, the use of natural diets such as castor leaves for rearing SfMNPV can greatly reduce the cost of production, although such systems are largely prone to contamination by extraneous viruses or microsporidians [4].

Not many studies have investigated the effect of cannibalism on baculovirus transmission in lepidopteran insects. Only some cannibalism-mediated insect–bacteria interactions were reported [45]. The current work demonstrates the effect of fall armyworm cannibalism on virus transmission. It was discovered that FAW cannibals were slow to attack their larval counterpart prey infected with high SfMNPV-KA1 PIB concentrations, meaning an existing trade-off by the cannibal caterpillars between starving and eating infected food. Moreover, the data established a significant interaction between prey body parts (head, abdomen, or tail) attacked and concentration of SfMNPV-KA1, which is evidence of the cannibal switching its decision to target a given prey body part based on the prey infection status. We were expecting that non-infected prey would be attacked at its tail because we assumed it is more vigorous and capable of using defense mandibles on the head, but the current results show that most of the cannibals targeted the heads of non-SfMNPV-KA1 infected prey. However, when infected with a high SfMNPV-KA1 concentration of 10^8^ PIB × mL^−1^ the preys were more frequently attacked at their tails. The most plausible explanation depicts existing exocrine chemical defense glands [46] at the vicinity of the larva’s tail. That gland secretion might be emitted by a vigorous non-infected larva. However, this is only speculative without clear empirical findings, and to the best of our knowledge, not many available reports support the argument. In any case, the highest SfMNPV-KA1 concentration in prey, the highest cannibal mortality, and the lowest cannibal pupa and adult numbers were associated on all observation dates. In addition to the horizontal effect of SfMNPV-KA1 caused by cannibalism, a vertical effect was discovered with a significant oviposition rate reduction in female cannibals followed by a low survival rate of their progeny. However, in relation to the mass production of the virus, cannibalism might compromise the yield of SfMNPV-based bioinsecticide [4]. Conversely, cannibalism could enhance baculoviral spread and natural control.

## 5. Conclusions

In conclusion, this work provides pioneering data on the virulence of the new SfMNPV isolate from Nigeria (SfMNPV-KA1), which proved more effective than its exotic counterpart from Argentina (SfMNPV-ARG). It revealed that host plant could play an important role in virus efficacy and demonstrated both horizontal and vertical effects of cannibalism in SfMNPV-KA1 transmission. Developments in in vitro multiplication of baculoviruses have been achieved, yet large-scale production of the viruses as commercial biopesticides has relied on in vivo multiplication in host insects [47]. One of the reasons is the significantly low cost involved and less technology-stringent production requirements for in vivo multiplication.

## Data Availability

The data presented in this study are available in Supplementary Material.

## Supplementary Materials

The following supporting information can be downloaded at: https://www.mdpi.com/article/10.3390/insects15040225/s1, Video S1: Fall armyworm cannibalism attack/defense fight (A), Fall armyworm cannibal searching for conspecific prey and attack/defense fight (B), Fall armyworm cannibal cutting conspecific prey head capsule (C), Fall armyworm cannibal consuming conspecific prey (D).
